# Insomnia: Risk Factor for Neurodegenerative Diseases

**DOI:** 10.7759/cureus.6004

**Published:** 2019-10-26

**Authors:** Sohaib A Shamim, Zain I Warriach, Muhammad Ali Tariq, Kiran F Rana, Bilal Haider Malik

**Affiliations:** 1 Neurology, California Institute of Behavioral Neurosciences and Psychology, Fairfield, USA; 2 Psychiatry, California Institute of Behavioral Neurosciences and Psychology, Fairfield, USA; 3 Internal Medicine, California Institute of Behavioral Neurosciences and Psychology, Fairfield, USA; 4 Family Medicine, California Institute of Behavioral Neurosciences and Psychology, Fairfield, USA

**Keywords:** dementia, alzheimer' disease, neurodegeneration, insomnia, non-restorative sleep, chronic short sleepers

## Abstract

Insomnia can be defined as difficulty falling asleep or maintaining sleep, waking up earlier than expected, or having non-restorative sleep. It is one of the most common sleep disorders in the world. Insomnia is a common symptom of many neurodegenerative diseases but only recently has it been found that it is a risk factor for neurodegenerative disorders such as Alzheimer's disease and Parkinson's disease.

We did a traditional review to analyze the relationship between insomnia and neurodegenerative diseases. We analyzed all the relevant articles on Pubmed and included studies done on humans over the last 10 years with full text available.

After reviewing the available literature on Pubmed, we conclude that insomnia is an important risk factor for neurodegenerative diseases. In addition, insomnia and neurodegenerative disorders have a complex and bi-directional relationship.

We think it requires further study to understand the sole contribution of insomnia to the development of various neurodegenerative diseases when different factors like mood problems, genetic factors, and environmental factors also contribute to the disease. It would also be advisable to use cognitive screening questionnaires in all sleep clinics in insomnia patients over 50 years of age to diagnose dementia early and to gather more sleep study data for prospective and retrospective research. The role of hypnotics in preventing neurodegenerative diseases through treating insomnia should also be assessed.

## Introduction and background

“The best cure for insomnia is to get a lot of sleep” - W. C. Fields.

The annual financial burden of the two most common neurodegenerative diseases in the U.S., Alzheimer’s disease and Parkinson’s disease, is about 277 billion and 25 billion, respectively [1-2]. The numbers of deaths from Alzheimer's disease and other dementia continue to rise every year despite advances in medical sciences, probably due to an aging population and with no definitive treatment or cure available to patients [2]. Therefore, it would be logical to study the risk factors leading to neurodegenerative diseases especially until some form of treatment is discovered. One of the most important and changeable risk factors leading to dementia later in life is poor sleep [3].

Insomnia is defined when a person has a hard time falling asleep or maintaining sleep, waking up earlier than expected, or having non-restorative sleep. Insomnia always has to be associated with some daytime sleepiness and fatigue [4]. A short sleeper is defined as a person who can only sleep less than seven hours on a nightly basis regardless of daytime symptoms [5-6]. Non-restorative sleep is when a person has sleep that does not restore the body despite having a normal sleep period, efficiency, and quality [7]. Insomnia, whether primary or secondary, is frequently associated with neurodegenerative processes. It has been found that insomnia and Alzheimer’s disease have a two-way relationship: Alzheimer’s disease causes sleep fragmentation while poor sleep leads to the increased deposition of β-Amyloid and hyper-phosphorylated Tau protein in the human brain and subsequent Alzheimer's disease [3]. It has also been described that insomnia in old age could lead to brain cell aging [8]. Insomnia also indirectly increases the risk of dementia by increasing the risk factors of dementia such as depression, hypertension, diabetes and obesity [9-13].

In this review article, we will try to summarize the data available from previous studies and prove if there is a strong link between insomnia and neurodegenerative disorders. We will describe the pathophysiologic and biochemical mechanisms through which insomnia leads to neurodegenerative changes in the brain. We will also give an account of the ways through which we can detect insomnia early on and decrease the morbidity and mortality of patients. A brief overview of polysomnography findings associated with insomnia and neurodegeneration will also be described. If insomnia is identified as an individual risk factor for neurodegenerative disorders, a new door of preventative and treatment strategies will open that could change the incidence and course of neurodegenerative disorders.

## Review

Methods

To analyze the association between insomnia and neurodegeneration, we organized a detailed review of published articles on Pubmed. Our search included articles containing regular keywords and Mesh keywords. We only included studies that had “full text” available. We excluded studies published before the last 10 years by applying the "10 years" filter and excluded studies that were done on animals and in languages other than English. PRISMA (Preferred Reporting Items for Systematic Reviews and Meta-Analyses) guidelines are not needed in a traditional review and, therefore, were not used. We did not use any quality assessment tools or quality appraisals. Statistical analysis was not needed in this traditional review and hence not done.

Results

Our results show that dementia and Alzheimer’s disease by far yielded the most number of research articles, with 66,157 and 48,236 studies. Neurodegeneration yielded 17,757 studies, insomnia yielded 8218, non-restorative sleep yielded 157, and chronic short sleepers yielded 51. Mesh keyword dementia yielded the maximum number of results with 41 studies, neurodegeneration came up with 11 studies, whereas insomnia yielded only three studies. About our actual topic, we searched “insomnia, neurodegenerative disorders,” and it resulted in 402 research papers. However, “sleep deprivation, insomnia” yielded only 63 results. The details of the results are given in Table 1 and Table 2.

**Table 1:** Regular Keywords

REGULAR KEYWORDS	DATABASE	NO. OF RESULTS
dementia	Pubmed	66157
Alzheimer’s disease	Pubmed	48236
neurodegeneration	Pubmed	17757
insomnia	Pubmed	8218
non-restorative sleep	Pubmed	157
chronic short sleepers	Pubmed	51

**Table 2:** Mesh Keywords

MESH KEYWORDS	DATABASE	NO. OF RESULTS
dementia	Pubmed	41
neurodegeneration	Pubmed	11
insomnia	Pubmed	3

Discussion

The link between insomnia and neurodegenerative diseases has recently been established. In this review article, we will summarize the work done in the literature available on Pubmed. A brief description of the polysomnography findings associated with insomnia and dementia will also be given.

Sleep Physiology and Biochemistry

The importance of sleep in human lives can be understood by the fact that a third of our time is spent during sleep and the productivity of the remaining two-thirds of our time depends on the quality of sleep we had. Sleep is defined as a reduction in the level of consciousness and body responses to external stimuli along with certain changes in brain electroencephalogram (EEG) [14]. Sleep has been classified into two types: rapid eye movement (REM) and non-REM (NREM) sleep. NREM sleep is further classified into three stages - N1, N2, and N3 - due to changes in the electrical activity of the brain [15].

Three main factors are controlling the normal sleep and wake pattern - intrinsic circadian rhythm, internal sleep homeostatic activity and external factors [16-17]. The circadian rhythm that conducts the everyday cycle of sleep and wakefulness is controlled by the suprachiasmatic nucleus (SCN) of the hypothalamus [18]. The circadian rhythm is mainly regulated by melatonin and light exposure, with melatonin rising a couple of hours before bedtime and promoting sleep, and light exposure reducing melatonin secretion and disrupting sleep [19]. Sleep homeostasis refers to the accumulation of sleep pressure as a response to prolonged wakefulness and the compensatory hypersomnia that occurs after sleep induction [15,18]. The interaction between sleep homeostasis and the SCN results in the regulation of sleep and wakefulness during the day [18]. At bedtime, the sleep homeostasis predominates and SCN output is decreased, thus promoting sleep, while in the morning, the SCN output increases with little or no sleep pressure, hence promoting alertness [18].

The major neurotransmitters promoting sleep include gamma-aminobutyric acid (GABA), galanin, and adenosine while the major neurotransmitters promoting wakefulness include orexin (hypocretin), histamine, acetylcholine, glutamate, norepinephrine, dopamine, and serotonin [18,20]. The ascending reticular activating system (ARAS) regulates alertness through a series of neuronal pathways that ascends from the brainstem and hypothalamus [18,21]. The lateral tegmental nuclei and pedunculopontine tegmental nuclei (LDT and PPT) of the brain stem use acetylcholine, the raphe nuclei use serotonin, the locus coeruleus uses epinephrine, and the tuberomammary nucleus of the hypothalamus uses histamine as the neurotransmitter to excite the cerebral cortex and are all necessary for maintaining arousal [21]. Sleepiness is mediated primarily by the ventrolateral preoptic nucleus (VLPO) of the hypothalamus via GABA and galanin [21].

Pathophysiology of Common Neurodegenerative Diseases

Neurodegenerative disease in the brain refers to the death of neurons that occurs through the course of many neurological diseases. The main mechanisms leading to neurodegeneration include abnormal protein accumulation and misfolding, oxidative stress, and mitochondrial dysfunction and neuroinflammation [22]. Alzheimer's disease is signified by the accumulation of β-Amyloid peptide and neurofibrillary tangles, which then lead to inflammation of neurons and cell death. Parkinson’s disease, which is the second most common neurodegenerative disease, is denoted by the deposition of alpha-synuclein and Lewy bodies in dopamine carrying neurons and subsequent neuron inflammation and cell death [23]. Amyotrophic lateral sclerosis, another neurodegenerative disease, is characterized by the accumulation of abnormal protein TDP-43 and the subsequent death of upper and lower motor neurons [24].

Insomnia and risk of Neurodegenerative Diseases

Insomnia is a common complaint among people with neurodegenerative diseases. The majority of neurodegenerative diseases are known to cause sleep disruption of some kind, but it has been only recently discovered that insomnia might be a risk factor for neurodegenerative diseases, such as Alzheimer’s disease, leading to the concept of a bi-directional relationship [3].

It has been shown in research studies that sleep contributes to the removal of harmful proteins such as β-Amyloid in brain cells [25]. Kang et al. (2009) showed in animal studies that chronic insomnia could lead to the accumulation of β-Amyloid protein in transgenic mice [3,26]. In human beings, Shokri-Kojori et al. (2018) demonstrated that sleep deprivation even for a single night leads to the increased deposition of β-Amyloid protein in brain cells [25,27].

Bubu OM et al. demonstrated in a meta-analysis that people with insomnia and other sleep problems had 1.68 (95% CI: 1.51-1.87) times more risk of developing dementia and/or Alzheimer's disease [28]. Lim AS et al. did a prospective study that demonstrated a 1.5 times increased risk of developing Alzheimer’s disease in patients with severe sleep fragmentation (90th percentile) as compared to patients with low sleep fragmentation (10th percentile) when followed for a period of six years [29]. Benedict et al. did a study in Sweden with a comparatively large sample size of 1574 in adults aged 50 years and older. They observed them for the next 40 years and measured the incidence of dementia overall and Alzheimer’s disease. They determined that people with a subjective complaint of insomnia had a 33% increased risk of dementia overall and a 51% increased risk of Alzheimer’s disease as compared to people without insomnia [30-31]. Hsiao et al. did a cohort study to assess the risk of Parkinson’s disease in people with non-apnea sleep disorders. They concluded that people with chronic insomnia were at the highest risk among other non-apnea sleep disorders for developing Parkinson’s disease in the future [32]. It has also been shown that insomnia can lead to depression and anxiety in Parkinson’s disease patients and vice versa and can also adversely affect the quality of life [33-34]. All these studies proved that insomnia might be a risk factor for dementia.

**Figure 1:**
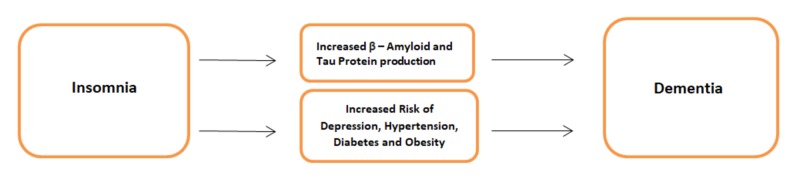
Insomnia As a Risk Factor for Dementia

Furthermore, it has been shown that patients who have an alteration of sleep architecture also have an increased risk of dementia. The decrease in REM sleep has been particularly linked to the increased incidence of dementia in humans [35]. Stage 3 sleep, which is also called slow-wave sleep or deep sleep, is of the utmost importance in patients with dementia. Slow-wave sleep is particularly important in memory consolidation and providing restorative sleep to individuals [36]. In the sleep research done at Washington University School of Medicine, it was demonstrated that people who have a reduced amount of Stage 3 sleep have an increased accumulation of hyperphosphorylated Tau proteins, which is another pathological hallmark of Alzheimer’s disease [36]. Also, aging and most neurodegenerative disorders are known to decrease the amount of REM sleep and slow-wave sleep, leading to a bi-directional relationship. The different ways through which insomnia leads to neurodegenerative diseases are summarized in Figure 1.

Table 3 below lists the important studies that are relevant to the review article.

**Table 3:** Important Studies That Are Relevant to the Review Article

No. of Studies	Author Name	Year of Publication	Country of Origin of the Study	Inference
1	Benedict C [33]	2015	Sweden	Insomnia is an important risk factor for dementia and Alzheimer's Disease.
2	Shi L [32]	2017	China	Insomnia increases the risk of Alzheimer’s Disease.
3	Shokri-Kojori E [29]	2018	USA	Acute Insomnia leads to Alzheimer’s Disease pathology (β-Amyloid accumulation) in the human brain.
4	Minakawa EN [3}	2019	Japan	Insomnia and dementia have a bi-directional relationship.

However, if there is such a strong link between insomnia and neurodegenerative diseases, it remains unclear why not all the patients with insomnia develop neurodegenerative diseases.

Limitations

Consistent with most researches, our research also has some limitations. Most research papers we studied had a small sample size and were not followed through the full course of the disease. The relevant research data about Alzheimer’s disease was abundant but was scarce about other neurodegenerative diseases such as Parkinson’s disease.

## Conclusions

After reviewing the available literature on PubMed, we conclude that the relationship between insomnia and neurodegenerative diseases is bi-directional and complex. We think that insomnia is an important risk factor for neurodegenerative diseases such as Alzheimer’s disease and Parkinson's disease. Chronic insomnia leads to neurodegenerative changes in Alzheimer’s disease brains through the accumulation of β-Amyloid and Tau proteins. However, it requires more research to evaluate if insomnia leads to an increased risk of other neurodegenerative diseases as well such as amyotrophic lateral sclerosis, frontotemporal dementia, etc. It requires further study to understand the sole contribution of insomnia to the development of various neurodegenerative diseases when different factors, such as like mood problems, genetic factors, and environmental factors play their role. Future studies should aim at comparing sleep studies of patients having different neurodegenerative diseases and looking for a common pattern in sleep architecture. It would also be advisable for all sleep clinics to use screening questionnaires, such as Montreal Cognitive Assessment (MoCA), in insomnia patients aged 50 years or older to detect early dementia and to gather more information about changes in sleep architecture with changes in MoCA score over time. Future research should also be done to assess the role of hypnotics that improve insomnia by increasing the level of slow-wave sleep and their role in preventing neurodegenerative diseases.
